# Development of polarity-reversed endometrial epithelial organoids

**DOI:** 10.1530/REP-23-0478

**Published:** 2024-02-15

**Authors:** Vakil Ahmad, Sai Goutham Reddy Yeddula, Bhanu P Telugu, Thomas E Spencer, Andrew M Kelleher

**Affiliations:** 1 Division of Animal Sciences, University of Missouri, Columbia, Missouri, USA; 2 Department of Obstetrics, Gynecology, and Women’s Health, University of Missouri, Columbia, Missouri, USA

## Abstract

**In brief:**

Polarity-reversed endometrial epithelial organoids exhibit histological and physiological characteristics resembling uterine epithelium *in vivo*, respond to hormones, and undergo secretory cell transformation. The ability to modify the polarity without impairing functionality, coupled with successful coculture with microbial and embryonic entities, paves the way for investigating complex interactions at the endometrial epithelial surface.

**Abstract:**

The uterine epithelium comprises a single layer of hormone-responsive polarized epithelial cells that line the lumen and form tubular glands. Endometrial epithelial organoids (EEO) can be generated from uterine epithelia and recapitulate cell composition and hormone responses *in vitro*. As such, the development of EEO represents a significant advance in facilitating mechanistic studies *in vitro*. However, a major limitation of the use of EEO cultured in basement membrane extract and other hydrogels is the inner location of the apical membrane (apical-in EEO), thereby hindering direct access to the apical surface of the epithelium to study interactions with the embryo or infectious agents such as viruses and bacteria. To address this challenge, we developed a suspension culture method to reverse the polarity of EEO. The result is an apical-out organoid that preserves a distinct apical–basolateral orientation and remains responsive to ovarian steroid hormones. Apical-out EEO were positive for the gland marker, FOXA2, and exhibited appropriate hormonal regulation of steroid hormone receptor expression. Notably, progesterone treatment resulted in secretory transformation in apical-out EEO, including a decrease in microvilli and cilia, and an increase in secretory granules. Likewise, reflective of *in vivo* conditions, ENPP3, a P4-regulated gene, was localized apically in steroid hormone-treated organoids. Coculture experiments with apical out EEO demonstrate the model’s utility in studying uterine epithelium interactions with bacteria (*E. coli*) and blastocysts. The apical out EEO model lays the foundation for developing new *in vitro* functional assays, particularly regarding epithelial interactions with embryos during pregnancy or other luminal constituents in a pathological or diseased state.

## Introduction

Understanding uterine epithelial biology is essential for improving women’s reproductive health, as defects in epithelial function contribute to various uterine pathologies and infertility (Burney & Giudice 2012, Strauss & Barbieri 2019, Wang *et al.* 2020, Gruber & Mechsner 2021). The uterine epithelium consists of a single layer of pseudostratified luminal epithelium and simple columnar glandular epithelium. The uterine epithelium has a critical role in maintaining the contents of the uterine lumen, facilitating blastocyst adhesion and attachment, and protecting the uterus from ascending infections (Aplin & Kimber 2004, Mitchell *et al.* 2015, Moreno *et al.* 2016, Benner *et al.* 2018, Kelleher *et al.* 2019). However, our understanding of the cellular and molecular mechanisms underlying endometrial epithelial physiology and function is limited due to the technical constraints of *in vitro* models and the ethical limitations of *in vivo* investigations, particularly during pregnancy.

Organoids have recently emerged as an indispensable model for studying epithelial biology, pathophysiology, and embryo–maternal interactions (Boretto *et al.* 2017, 2019, Turco *et al.* 2017, Fitzgerald *et al.* 2019, Fitzgerald *et al.* 2021, Murphy *et al.* 2022). To establish endometrial epithelial organoids (EEO), epithelial cells from the endometrium are isolated through enzymatic digestion and mechanical disruption. These cells are embedded within a basement membrane extract (BME) and form three-dimensional structures. Growth media supplemented with a cocktail of growth factors promotes cell proliferation, differentiation, and self-organization into mature organoids (Boretto *et al.* 2017, Turco *et al.* 2017). Importantly, EEO can be propagated while retaining key features of the endometrial epithelium, including tissue organization, cellular composition, gene expression signatures, steroid hormone response, and protein secretion profiles (Boretto *et al.* 2017, Turco *et al.* 2017, Fitzgerald *et al.* 2019, 2021, 2023). Thus, EEO can serve as a powerful *in vitro* model with significant potential for addressing critical questions in reproductive biology related to epithelial function, regeneration, and development.

A defining feature of BME-embedded three-dimensional organoid structures is a polarized epithelium with a central lumen. The apical-in/basal-out polarity of EEO presents a challenge to studying epithelial interactions with embryos or infection by microbes or viruses that reside in the uterine lumen (Boretto *et al.* 2017, 2019, Co *et al.* 2019, Simintiras *et al.* 2021, Kakni *et al.* 2023, Wijesekara *et al.* 2023). Indeed, access to the apical surface of BME embedded organoids is restricted, and microinjection techniques are required to deliver or to remove factors from the apical surface or lumen of the organoids (Bartfeld & Clevers 2015, Bartfeld *et al.* 2015, Co *et al.* 2019, Simintiras *et al.* 2021). This technique can be technically challenging, requires access to specialized equipment, and necessitates organoid growth to large diameters, thus limiting the utility of current EEO models. To overcome this limitation, a suspension culture method to reverse EEO polarity was developed based on methods reported for human enteroid and airway organoids (Co *et al.* 2019, 2021, Kakni *et al.* 2023, Wijesekara *et al.* 2023). Our studies demonstrate that removing the BME and subsequent suspension culture of EEO in low-attachment plates alters the polarity of EEO without compromising their physiological hormone response and viability. The polarity-reversed EEO exhibit histological and physiological characteristics resembling uterine epithelium *in vivo*, respond to hormones, and undergo secretory cell transformation. Coculture experiments further reveal the preferential interaction of microbes with polarity-reversed (apical-out; AO) organoids, providing a more representative model of ascending infection *in vivo*. Likewise, coculture with blastocysts demonstrates the hormonal impact on EEO–embryo interactions. Taken together, the apical-out EEO can be utilized for a broad range of applications beyond the scope of this initial characterization, including studies on epithelial–microbial interactions, drug screening, and investigations into uterine epithelial–embryo interactions during implantation.

## Methods

### Endometrial epithelial organoid establishment and maintenance

Endometrial epithelial organoids (EEO) were isolated, cultured, and maintained as previously described (Turco *et al.* 2017, Fitzgerald *et al.* 2019, 2023). Primary human endometrial epithelial cells were isolated from endometrial biopsies of premenopausal women with no history of endometrial pathology or endocrine disorders. All donors were not taking any steroid-modulating medications. Written informed consent was obtained from each donor and the study was approved by the University of Missouri Institutional Review Board (IRB Project Approval Number: 2011513). Those cells were washed and subsequently resuspended in Cultrex BME (Cat. No. 343300501, R&D Systems) and plated in 20–25 µL droplets of Cultrex. The droplets were solidified by incubation at 37°C for 15 min before being overlaid warm organoid expansion culture medium as reported previously (Fitzgerald *et al.* 2019). To passage, organoids were released from Cultrex by repetitive pipetting in cold PBS using a 1 mL pipette tip and transferred to a 15 mL conical tube. After centrifugation at 300 ***g*** for 5 min at 4°C, the supernatant was removed, and the pellet was resuspended by repeated pipetting with 1 mL Advanced DMEM/F12 (Cat. No. 12634010, Gibco). The cells were washed with an additional 1 mL of Advanced DMEM/F12 prior to resuspension in 80% Cultrex and 20% growth media. All organoid characterization experiments were conducted using 2–4 independent donors and repeated using separate passages containing three technical (well) replicates.

### Generation and propagation of apical-out EEO

To generate AO EEO, the growth medium was aspirated and Cultrex droplets were gently dislodged into the cell recovery solution (Cat. No. 354270, Corning) and transferred to Eppendorf tubes using wide-bore 1 mL pipette tips. The tubes were rotated gently for 1 h at 4°C. The organoids were pelleted down by centrifugation at 20 ***g*** for 3 min and washed with Advanced DMEM/F12. Finally, organoids were resuspended in an expansion medium supplemented with the ROCK inhibitor Y27632 (Cat. No. 1293823, PeproTech) and seeded as a suspension culture in ultralow-attachment plates (Cat. No. 3473, Corning). The suspension culture was gently agitated twice daily using a pipette to avoid clumping of organoids. The growth medium was replaced every other day, a process that involved transferring the organoids to a tube using wide-bore pipette tips, allowing them to settle for 5 min (or alternatively, centrifuging at 20 ***g*** for 30 s), aspirating the supernatant, resuspending the organoid pellet in growth medium, and seeding them in ultralow-attachment plates.

### Immunofluorescence analysis

For immunofluorescence staining of organoids embedded in Cultrex, the expansion medium was aspirated and EEO were fixed using 2.5% paraformaldehyde (Cat. No. 15710, Electron Microscopy Sciences) for 20 min at room temperature (RT), followed by staining with hematoxylin solution (Cat. No. HHS32, Sigma-Aldrich) for 10 min at RT. After washing with PBS, droplets were embedded in 2% low-melting point agarose (Cat. No. 9012366, Fisher Scientific) and allowed to solidify at RT for 10 min and then at 4°C for 1 h. Droplets embedded in agarose were transferred from wells using a flat spatula and placed in cassettes for paraffin embedding and sectioning (5 µm). Suspension culture EEO were transferred to an Eppendorf tube and centrifuge at 20 ***g*** for 30 s. The supernatant was aspirated and EEO were resuspended in 2.5% paraformaldehyde for 20 min, followed by staining with hematoxylin solution for 10 min at RT. After washing with PBS, organoids were transferred to plastic molds using wide bore pipet tip, excessive PBS was aspirated and organoids were embedded in 2% low-melting point agarose and allowed to solidify at RT for 10 min and then at 4°C for 1 h. Organoids embedded in agarose were placed in cassettes for paraffin embedding and sectioning (5 µm). All sections were mounted on slides, baked at 60°C for 30 min, deparaffinized in xylene, and rehydrated in a graded alcohol series. Deparaffinized sections were subjected to antigen retrieval by incubating sections in Tris-EDTA (Cat. No. 93684; Abcam) at 95°C. All slides were blocked with 5% (v/v) normal goat serum (NGS) (Cat. No. 016201, Thermo Fisher Scientific) in PBS at RT for 1 h and incubated with primary antibodies (PGR – Cell signaling Technology 8757S; ESR1 – Cell Signaling Technology 8644S; ZO1 - Invitrogen 33-9100, GFP – Abcam ab5450, ENPP3 – Sigma-Aldrich HPA043772, KI67 – Abcam ab15580) overnight at 4°C in 1% (v/v) NGS diluted in PBS. Immunofluorescence visualization was performed with (Molecular Probes Alexa Fluor 660 Phalloidin – Thermo Scientific A22285, beta-catenin – Abcam 32572) Alexa 488 or 590 or 647-conjugated secondary antibodies (1:500 dilution; Cat. Nos. 112545143; 111585144; 111605144, Jackson ImmunoResearch). Sections were counterstained with Hoechst 33342 (2 μg/mL dilution; Cat. No. H3570; Invitrogen) before affixing coverslips with ProLong™ Diamond Antifade Mountant (Cat. No. 36961; Invitrogen). Images were taken with a Leica DM6 B upright microscope and Leica K8 camera using Leica Application Suite X (LAS X).

### Hormone treatment

AO or AI EEO were either plated in Cultrex droplets or seeded as a suspension culture in ultralow-attachment plates. Both embedded or suspension culture organoids were grown for 4 days after passaging and then treated with either vehicle as a control (100% ethanol) or 10 nM estradiol-17β (E2; Cat. No. E1024; Sigma) for 2 days. Next, organoids were treated with either vehicle control or 10 nM E2 and 1 mM medroxyprogesterone acetate (Cat. No. PHR1589; Sigma) for 6 days with media changed every 2 days. Each treatment was performed in triplicate wells, and organoids derived from two individual donors were used. Following treatment, the organoids were harvested for immunofluorescence, transmission and scanning electron microscopy (SEM).

### Transmission and scanning electron microscopy

All reagents were purchased from Electron Microscopy Sciences and all specimen preparation was performed at the Electron Microscopy Core of the University of Missouri. Organoids were fixed in 2% paraformaldehyde, 2% glutaraldehyde in 100 mM sodium cacodylate buffer, pH 7.35. Fixed organoids were rinsed with 100 mM sodium cacodylate buffer, pH 7.35 containing 130 mM sucrose. Secondary fixation was performed using 1 % osmium tetroxide (Ted Pella, Inc. Redding, CA, USA) in cacodylate buffer and incubated at 4°C for 1 h, then rinsed with cacodylate buffer, and further with distilled water. A graded dehydration series was performed using ethanol. Specimens were dried using the Tousimis Autosamdri 815 Critical Point Dryer (Tousimis, Rockville, MD, USA) and finally sputter coated with 10 nm of platinum using the EMS 150T-ES Sputter Coater. Images were acquired with a FEI Quanta 600F scanning electron microscope (FEI, Hillsboro, OR, USA). For TEM, fixed organoids were resuspended in HistoGel (Thermo Scientific). Next, fixed organoids were rinsed with 100 mM sodium cacodylate buffer, pH 7.35 (Sigma-Aldrich) and 130 mM sucrose. Each sample was allowed to settle, and the resulting pellet was resuspended in HistoGel (Thermo Scientific). Next, fixed cells were rinsed with 100 mM sodium cacodylate buffer, pH 7.35 containing 130 mM sucrose. Secondary fixation was performed using 1% osmium tetroxide (Ted Pella, Inc. Redding, CA, USA) in cacodylate buffer. Specimens were next incubated at 4°C for 1 h, then rinsed with cacodylate buffer and further with distilled water. En bloc staining was performed using 1% aqueous uranyl acetate and incubated at 4°C overnight, then rinsed with distilled water. A graded dehydration series was performed using ethanol, transitioned into acetone, and dehydrated tissues were then infiltrated with EMbed 812 resin and polymerized at 60°C overnight. Sections were cut to a thickness of 75 nm using an ultramicrotome (Ultracut UCT, Leica Microsystems) and a diamond knife (Diatome, Hatfield, PA, USA). Images were acquired with a JEOL JEM 1400 transmission electron microscope (JEOL, Peabody, MA, USA) at 80 kV on a Gatan Rio CMOS camera (Gatan, Inc, Pleasanton, CA, USA). ImageJ (version 2.3.0/1.53q) was used for quantification of microvilli and cilia from three randomly selected organoids in each group.

### Coculture of organoids with bacteria

*Escherichia coli* GFP (ATCC 25922 strain) were cultured overnight in LB broth. Bacteria were pelleted by centrifugation at 15,000 ***g*** for 10 min at 4°C, followed by two washes with ice-cold PBS. Finally, the bacterial cells were resuspended in 1 mL of organoid growth medium.

EEO were released from the Cultrex BME using the cell recovery solution, washed with Advanced DMEM/F12, and then resuspended in the organoid expansion medium. EEO were seeded in either ultralow-attachment plates to generate AO organoids or re-embedded in Cultrex BME to grow AI organoids. The organoids were allowed to grow for 4 days. Subsequently, EEO were mixed with bacterial cells by rotating for 1 h in a tissue culture incubator. AI EEO were released from BME as described above prior to bacterial incubation. Following incubation, excess bacterial cells were removed by thorough washing with PBS. AI organoids were re-embedded in Cultrex BME, while AO organoids were cultured in ultralow-attachment plates. The organoids were allowed to recover for 24 h prior to live imaging using a Leica DMi8 imaging system every 48 h. The growth medium was replenished every other day. Bacterial GFP intensity was quantified using image J (version 2.3.0/1.53q) freehand selection. Area was marked around the organoid and mean fluorescence intensity was measured. A similar region was selected next to the organoid with no blastocyst present to calculate the background fluorescence. Corrected GFP intensity was calculated by subtracting the background from organoid intensity.

### Coculture of organoids with mouse blastocysts

Organoids were released from the Cultrex BME using the cell recovery solution, washed with Advanced DMEM, resuspended in the organoid expansion medium, and then seeded onto ultralow-attachment plates to generate AO EEO as described above. Organoids were treated with either vehicle or E2 for 2 days and E2 + MPA for an additional 6 days prior to the addition of blastocyst to the culture system. Blastocysts were collected from 4–6-week-old C57BL/6-Tg(UBC-GFP)30Scha/J mice (RRID: IMSR_JAX:004353). The mice were superovulated by intraperitoneal administration of 5 IU of PMSG followed by 5 IU of hCG after 46-48 h (Johnson *et al.* 1998). After hCG injection, the mice were placed with fertile males of the same strain for overnight mating. Mice with copulatory plugs were humanely euthanized 2.5 days later, and oviducts were collected into the M2 medium (Cat. No. MR-015-D, Millipore Sigma) for embryo recovery. The collected embryos were washed with preequilibrated KSOM media (Cat. No. MR-106-D, Millipore Sigma) and cultured until day 4.5. Any blastocysts that were still in zona pellucida were subjected to an acid Tyrode solution (Cat. No. MR-004-D; Millipore Sigma) to remove the zona (Hirai *et al.* 2011). Blastocysts were then washed with KSOM media, and 15-20 blastocysts were cocultured with ~50 vehicle-treated or hormone-treated AO or AI organoids from two independent donors in organoids base media supplemented with either vehicle or 10 nM E2 and 1 mM MPA at 37°C, 5% CO_2_, 5% O_2_ for 24 h. Coculture was mixed using wide-bore pipet tip 10 times immediately prior to live imaging using a Leica DMi8 microscope. The total number of GFP blastocysts attached were counted.

### Statistical analysis

The data are presented as the mean ± s.d., as determined from at least three independent experiments. All organoid characterization experiments were conducted using 2–4 independent donors and repeated using separate passages containing three technical (well) replicates. Following the Shapiro–Wilk test for normality, the data were analyzed using Student’s *t*-test (GraphPad Prism 9). A *P*-value of less than 0.05 was considered statistically significant.

## Results

### Development of endometrial epithelial organoids

**Figure 1:**
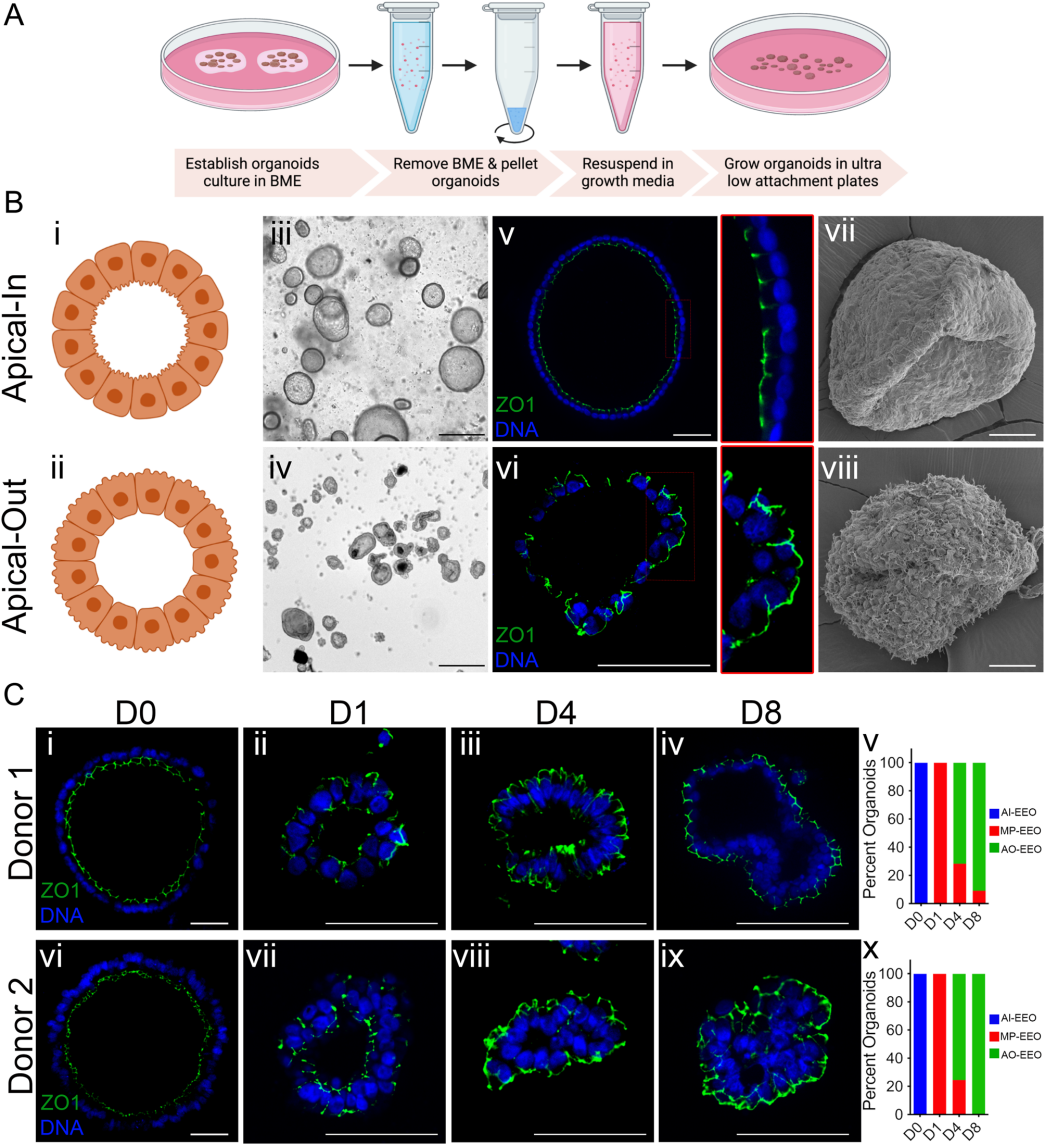
Generation and characterization of apical-out organoids. (A) Schematic of approach to generate apical-out endometrial epithelial organoids (AO-EEO or AO); (B) Characterization of AO. (i–ii) cartoons of epithelial cells in apical-in organoids (AI) exhibiting apical surface facing the lumen or in AO exhibiting apical surface exposed to the outer environment; (iii–iv) bright field images of organoids grown in Cultrex BME or in suspension culture for eight days showing gross morphology; scale bars, 100 µm; (v–vi) Immunofluorescence staining of apical surface marker zonula occludens 1 (ZO1) on paraffin sections of AI or AO; scale bars, 20 µm and 60 µm for v and vi respectively. Sections were counterstained with Hoechst to detect DNA; Areas outlined in red boxes in v and vi magnified to demonstrate apical and basal surfaces, separated by vertical line; (vii–viii) scanning electron microscopy (SEM) images of AI or AO showing surface microarchitecture; scale bars, 50 µm. (C) Time-dependent screening of organoids flipping from day 0 to day 8 detected by ZO1 immunofluorescence (i–iv and vi–ix), categorized as apical-in, apical-out, or mixed polarity and quantified in (v, x); scale bars, 50 µm (i, vi) and 150 µm (ii–iv and vii–ix). Schematic in A created with Biorender.com.

The current EEO model poses challenges in studying experimental interactions between the epithelial surface and luminal contents, such as during embryo–uterine interactions and microbial infections. Building on the concept of polarity reversal observed in organoids derived from the gastrointestinal tract (Co *et al.* 2019, 2021, Kakni *et al.* 2023, Wijesekara *et al.* 2023), we determined if the polarity of EEO could be reversed by chemically releasing intact organoids from the basement membrane extract (BME) and subsequently cultivating them in suspension culture. These studies employed EEO from the endometrium of premenopausal healthy women that were cultured in BME under WNT activating growth conditions as described previously (Boretto *et al.* 2017, 2019, Fitzgerald *et al.* 2021) (Fig. 1A). The human EEO formed spherical structures with the basolateral epithelial surface facing outward in contact with the BME, while the apical surface was oriented toward the inside of the organoid (Fig. 1B). To maintain the integrity of EEO during removal from BME, cell recovery solution was used to depolymerize the BME and release organoids without disrupting the organoid three-dimensional structure. The EEO were then transferred to suspension culture in growth media using ultralow-attachment plates (Fig. 1A). Visible changes in the gross morphology of EEO were observed in suspension culture, but both BME-embedded and suspension cultured EEO maintained a single-layered epithelium with a central lumen (Fig. 1B; iii–vi). Immunofluorescence imaging of apical surface proteins zonula occludens 1 (ZO1), F-actin, as well as the lateral and basement membrane-associated protein beta-catenin (CTNNB1), revealed that EEO in suspension culture exhibited reversal of apical–basal polarity after several days in culture, resulting in an outward-facing apical surface (referred to as apical-out EEO or AO). In contrast, BME-embedded organoids (referred to as apical-in EEO or AI) displayed the expected apical surface facing the central lumen (Fig. 1B; v, vi, Supplementary Fig. 1A, B, and E, see section on supplementary materials given at the end of this article). Scanning electron microscopy (SEM) further confirmed the polarity reversal with the outward apical surface covered with microvilli in suspension cultured organoids, while the outward surface of AI organoids was smooth (Fig. 1B; vii, viii). Importantly, the viability and growth of EEO in suspension culture was not altered with similar numbers of proliferating cells based on Ki67 immunostaining compared to BME-embedded EEO (Supplementary Fig. 1C). Consistent nuclear FOXA2 was observed in cells within the EEO; however, not all cells within a single organoid were FOXA2 positive, signifying they are a mixture of luminal and glandular epithelial (Supplementary Fig. 1D).

To understand the kinetics of EEO polarity reversal, immunofluorescent microscopy was used to quantify the percentage of EEO exhibiting apical-in, apical-out, or mixed (partial apical-in and apical-out) polarity in a time course experiment. EEO obtained from independent donors were released from BME, suspension cultured, and evaluated over 8 days (Fig. 1C and Supplementary Fig. 1E). ZO1 localization revealed the reversal of apical–basal polarity commenced within 24 h of the initiation of suspension culture, with all EEO displaying mixed polarity after day 1 (Fig. 1C; i, ii, vi, vii, v, x). On day 4 of suspension culture, the majority (>70%) of EEO exhibited apical-out polarity (Fig. 1C; iii, viii, v, x). On day 8, over 90% of EEO demonstrated AO polarity (Fig. 1C; iv, ix, v, x). Scanning electron microscopy (SEM) analysis found that microvilli emerged on the outer surface of EEO cultured in suspension by day 1 with increased abundance observed on days 4 and 8 (Supplementary Fig. 1F). Of note, the polarity reversal method was also successful with mouse EEO, resulting in complete polarity reversal in suspension culture (Supplementary Fig. 1G). These results indicate that EEO polarity reversal can be reliably achieved within 1 week by removing BME and performing suspension culture.

### Apical-out EEO exhibit physiological hormone responsiveness

**Figure 2:**
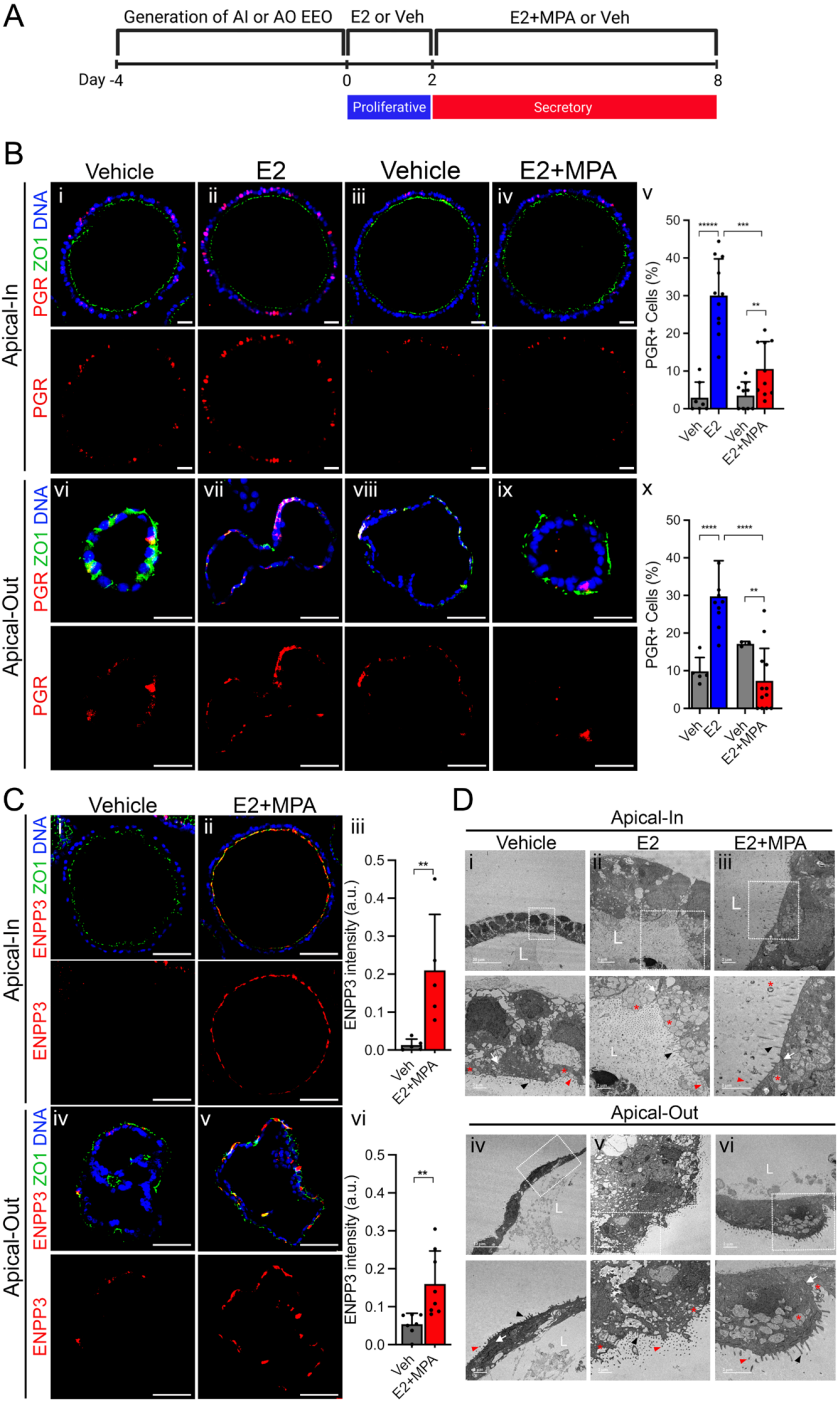
Apical-out organoids show similar response to uterine hormones as apical-in organoids. (A) Schematic showing the experimental design to treat AI or AO organoids with steroid hormones. (B) Representative images of immunofluorescence staining of PGR (red) and ZO1 (green) on paraffin sections of AI or AO treated with vehicle or E2 for 2 days and/or E2 + MPA for 6 days. Organoids were counterstained with Hoechst (blue) (i–vi and vi–ix); scale bars, 20 µm (i–vi) and 40 µm (vi–ix). Quantification of PGR-positive cells in AI and AO (v and x); (C) Representative images of immunofluorescence staining of ENPP3 (red) and ZO1 (green) on paraffin sections of AI or AO organoids treated with vehicle or E2 for 2 days and then with E2 + MPA for 6 days. Organoids were counterstained with Hoechst (blue). All scale bars in C are 40 µm (i–ii and iv–v). Quantification of ENPP3 (red) signal intensity (iii, vi). Bar graphs in B and C represent mean ± s.d. Solid dots represent the average organoid per biological replicate. **P < 0.01, ***P < 0.001, ****P < 0.0001 (Student’s t test). (D) Representative TEM images of AI or AO organoids treated with vehicle, E2 or E2 + MPA. Areas outlined in boxes are magnified in panels below to demonstrate the distribution of cilia (red filled triangles), microvilli (black filled triangles), tight junctions (white arrow), and secretory vacuoles (red asterisk) on the apical surface. Letter ‘L’ indicates the lumen of organoids. Scale bars in D are indicated within each image. Schematic in A created with Biorender.com.

EEO mimic physiological responses to progesterone including downregulation of PGR (progesterone receptor) expression and upregulation of progesterone-responsive genes (Turco *et al.* 2017, Fitzgerald *et al.* 2019, 2021, 2023). To assess the impact of polarity reversal on EEO response to steroid hormones, AI and AO EEO were treated with E2 followed by E2 + MPA (Fig. 2A) as previously described (Fitzgerald *et al.* 2019, 2023). MPA is non-metabolizable PGR agonist. Both AI and AO responded to steroid hormone treatment, and no differences were observed in the number of PGR-positive and ESR1-positive cells between AI and AO organoids (Fig. 2B and Supplementary Fig. 2A). Treatment with E2 alone increased the number of PGR positive cells, which was attenuated by MPA in both AO and AI EEO (Fig. 2B). Treatment with E2 also increased the number of ESR1 positive cells and increased the number of proliferative Ki67-positive cells in both AO and AI EEO compared to the control (Supplementary Fig. 2A and B). The organoid response to steroid hormone treatment is consistent with previous reports and reflects the *in vivo* response to E2 and progesterone (Lessey *et al.* 1988, Fitzgerald *et al.* 2019, 2023).

Ectonucleotide pyrophosphatase (ENPP3) is a progesterone responsive gene in the human endometrium and EEO (Boggavarapu *et al.* 2016, Fitzgerald *et al.* 2023). ENPP3 was substantially increased in both AO and AI EEO treated with E2 and MPA (Fig. 2C). Immunoreactive ENPP3 was observed on the apical surface of the EEO, reflecting the *in vivo* secretion pattern of ENPP3 and the polarity reversal phenotype of organoids cultured in suspension, further demonstrating the progesterone response of the AO organoids (Fig. 2C). The apical surface of uterine epithelial cells undergoes significant changes in response to steroid hormones and preparation for implantation (Murphy 2004). Transmission Electron Microscopy (TEM) analysis of E2-treated organoids revealed an increase in the abundance of cilia and length of microvilli, which are both reduced by concurrent MPA treatment (Fig. 2D and Supplementary Fig. 2C). Likewise, treatment with E2 + MPA resulted in the accumulation of secretory vacuoles at the apical surface of both AI and AO EEO (Fig. 2D). Collectively, these results support the idea that AO EEO maintain their physiological responses to steroid hormones.

### Apical-out EEO for the study of cell interactions

**Figure 3:**
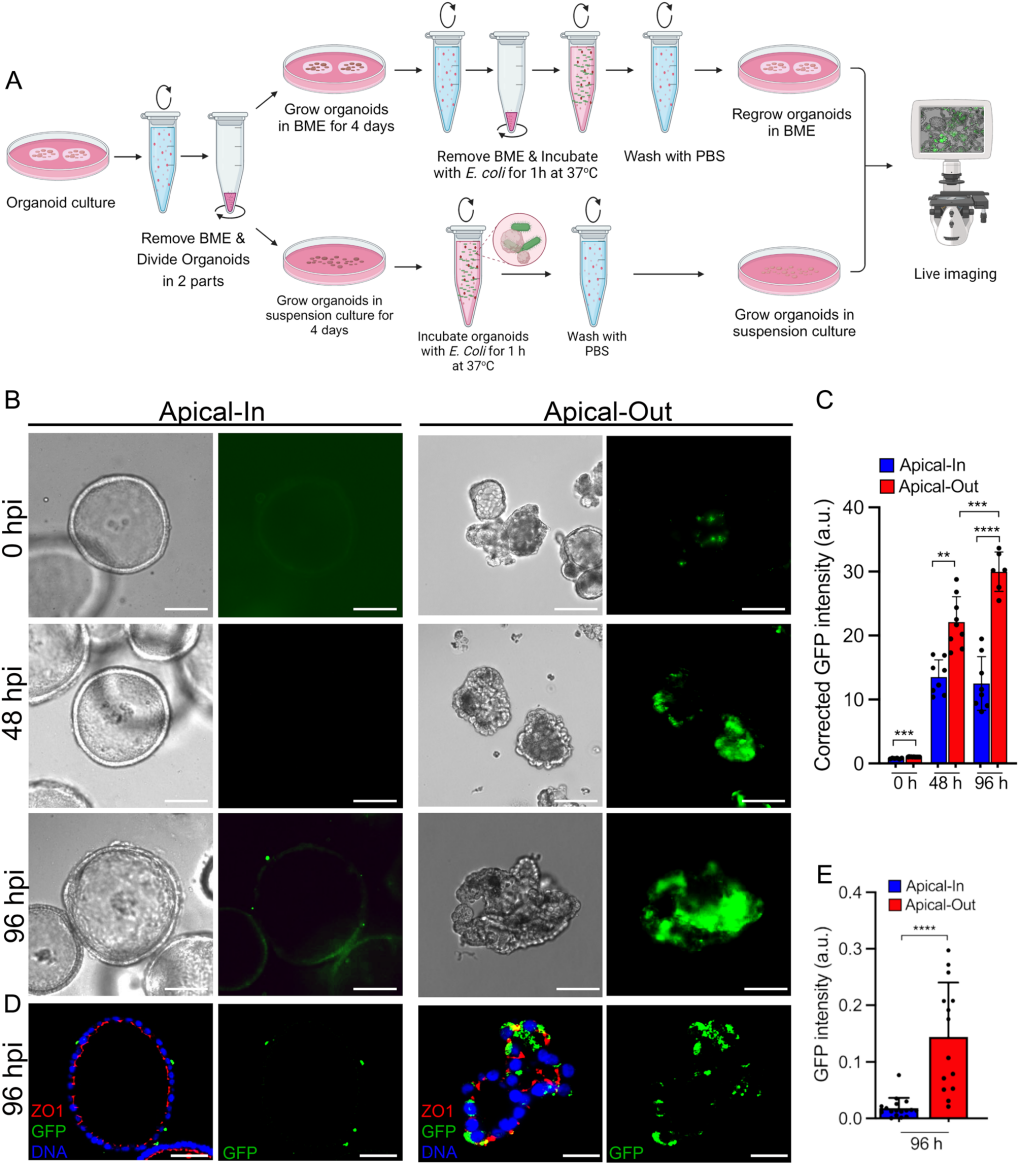
Apical-out organoids show susceptibility to *E. coli* infection. (A) Schematic showing the experimental design to infect AI or AO with *E. coli^GFP^*. (B) Bright field images from live epifluorescence imaging of AI or AO organoids infected with GFP-labeled *E. coli^GFP^* for 0 hpi, 48 hpi, and 96 hpi; All scale bars are 100 µm. (C) Quantification of GFP fluorescence intensity. (D) Representative images of immunofluorescence staining of ZO1 (red) and GFP (green) using antibodies against GFP from AI or AO infected with GFP-labeled *E. coli* for 96 h. Organoids were counterstained with Hoechst to detect DNA; All scale bars, 40 µm. (E) Quantification of GFP fluorescence intensity. Plots in C and E represent mean ± s.d. Solid dots represent the average GFP intensity per biological replicate. **P < 0.01, ***P <0.001, ****P < 0.0001 (Student’s *t*-test). Schematic in A created with Biorender.com.

EEOs offer significant potential for studying the interactions between the epithelium and components of the uterine lumen. However, structural and technical limitations have hindered investigations in this area. Ascending *E. coli* infection from the vagina to the uterus is a significant cause of disease in both pregnant and nonpregnant women (Brunham *et al.* 2015). In order to establish effective coculture systems for future mechanistic studies, interactions of AO EEO with a fluorescently labeled reporter strain of *E. coli* were studied (Fig. 3A). An increased attachment and replication of* E. coli^GFP^* (ATCC #25922) was observed in AO organoids compared to AI organoids at 0 and 48 h post infection (Fig. 3B, C, and D). Extended culture for 96 h post infection did not result in evident changes in the morphology of AO EEO; however, a steady increase in GFP intensity was observed, indicating bacterial replication (Hirako *et al.* 2022). In contrast, the GFP intensity remained low and unchanged in AI organoids after 48 and 96 h post infection (Fig. 3B, C, and D).

**Figure 4:**
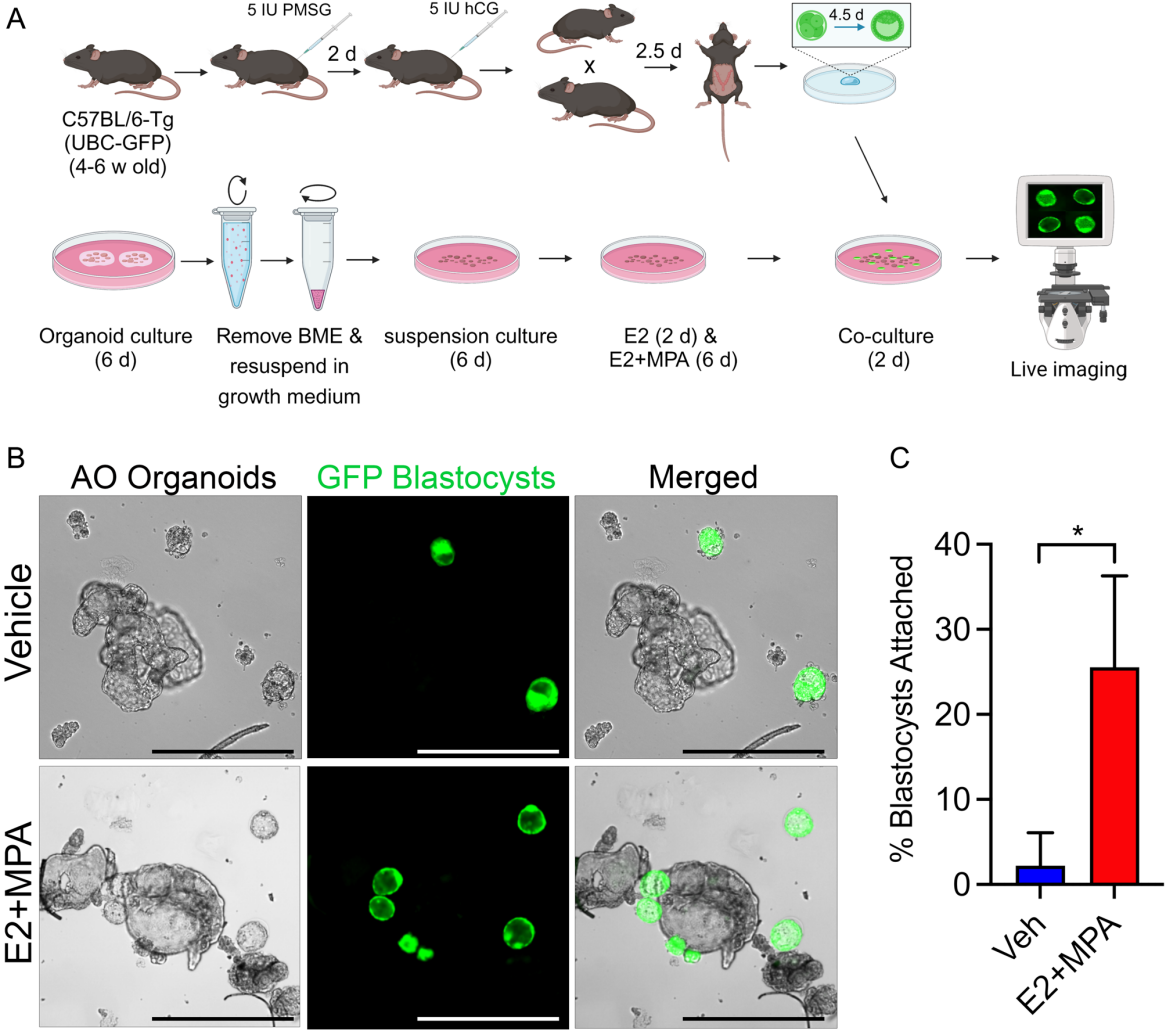
Apical-out organoids attach with blastocysts. (A) Schematic showing experimental design to coculture of mouse blastocysts and organoids. (B) Bright field, GFP epifluorescence and merged images of AO treated with vehicle or E2 for 2 days and E2 + MPA for 6 days, and then cocultured with mouse GFP labeled blastocysts for 24 h. scale bars, 100 µm. (C) Quantification of mouse blastocysts attached with vehicle or hormone treated AO organoids from three independent trials.

Finally, the coculture of blastocysts with AO EEO in suspension culture demonstrated the potential for studying endometrial–embryo interactions without using BME (Fig. 4A). AO were cultured in ultralow-attachment plates with implantation-competent mouse blastocysts expressing GFP (RRID: IMSR_JAX:004353) to evaluate the impact of steroid hormone treatment on EEO–embryo attachment. Blastocysts did not attach to vehicle treated AO EEO, whereas blastocysts did attach to AO EEO treated with a steroid hormone regimen mimicking the mid-secretory phase (Fitzgerald *et al.* 2019, 2023) (Fig. 4B and C). Blastocysts were not cultured with AI organoids as the interactions of the trophoblast cells with the basement membrane of EEO are not physiologically relevant. These findings confirm the ability of hormonally primed organoids to interact with and support the attachment of blastocysts (Rawlings *et al.* 2021), suggesting that this model could be an excellent tool for studying endometrial embryo interactions.

## Discussion

EEO are having a major impact on *in vitro* models of the uterus. These organoids have found broad application in studies pertaining to epithelial development, physiology, and disease due to their ability to mimic the properties of the uterine epithelium *in vivo*. Original reports and subsequent studies highlight EEO have a self-organized apicobasal structure, where the basal surface contacts the BME and the apical side is inside forming a closed lumen. Thus, access to the apical surface of AI EEO is restricted and microinjection techniques are required to deliver or to remove factors from the apical surface or lumen of the organoids (Bartfeld & Clevers 2015, Bartfeld *et al.* 2015, Co *et al.* 2019, Simintiras *et al.* 2021). This technique can be technically challenging due to the requirement for organoid growth to large diameters and potential contamination of the apical organoid compartment. Moreover, normalizing the exposure of microinjected experimental agents into the organoid lumen is complex due to the small volumes and variability in organoid size.

Using techniques from other organoid systems (Co *et al.* 2019, Giobbe *et al.* 2021, Nash *et al.* 2021) polarity reversal was achieved with EEO from the human and mouse endometrium by removing BME and performing suspension culture for 1 week. In the absence of BME and in suspension culture, EEO undergo epithelial rearrangement in which the apical surface everts to give rise to inside-out polar organization. During the reversal process, epithelial cells are shed from the AO resulting in organoids with decreased area. A similar process of epithelial eversion is observed in porcine thyroid follicles and gut spheroids in suspension culture (Graebert *et al.* 1997, Co *et al.* 2019). Other mechanisms of polarity reversal include the redistribution of basolateral proteins and migration of organelles from one epithelial pole to the other (Wang *et al.* 1990, Graebert *et al.* 1997, Co *et al.* 2019). The apical-out EEO model recapitulates critical functions of the endometrial epithelium *in utero*. Similar to apical-in EEO, apical-out EEO, despite the physical reorientation, retained their structural integrity and inherent functionality, including proliferation and steroid hormone receptor expression and response (Boretto *et al.* 2017, Turco *et al.* 2017, Boretto *et al.* 2019, Fitzgerald *et al.* 2019, 2023). Thus, polarity-reversed EEO can withstand structural manipulation without compromising their biological properties, maintaining their usefulness in exploring endometrial physiology and pathophysiology (Boretto *et al.* 2017, 2019, Turco *et al.* 2017, Fitzgerald *et al.* 2021). The ability to form these organoids with an apical-out orientation is expected to enhance their application in research that requires interaction between the luminal epithelium with embryos as well as microbes and viruses (Fitzgerald *et al.* 2021, Simintiras *et al.* 2021, Murphy *et al.* 2022).

To test the utility of the apical-out EEO model against the traditional apical-in model, a system was devised to investigate apical endometrial interactions with *E. coli* and mouse blastocysts. These serve as representative models for pathological and physiological interactions of the endometrial epithelium, respectively. The ability of the apical-out organoids to establish functional connections with these external entities suggests their potential use in investigating diverse interactions occurring within the uterine lumen. Specifically, the enhanced attachment and replication of *E. coli* on the accessible apical surface in suspension culture organoids present a useful model for studying ascending microbial infections. Attachment to epithelial cells is an early key phase in the pathogenesis of many bacterial pathogens, including *E. coli* (Kalita *et al.* 2014), which determines the infectious potential of these pathogens. Ascending *E. coli* infection from the vagina to the uterus is a significant cause of disease in both pregnant and nonpregnant women (Brunham *et al.* 2015). During pregnancy, *E. coli* infection can result in multiple negative consequences for the mother and child, such as neonatal or maternal sepsis, miscarriage, and preterm birth (Cools 2017).

The direct study of human embryo implantation is challenging due to ethical concerns and technical barriers, and animal models do not perfectly mirror the human process. The successful coculture and enhanced attachment of mouse blastocysts with apical-out organoids treated with steroid hormones marks an advancement in the potential to model *in vitro* endometrial–embryo interactions to study early pregnancy loss. Future studies may implement a coculture system containing AO EEO, stromal cells, and trophoblast organoids to study cell–cell interactions at the maternal–fetal interface.

In summary, a validated method was developed here to generate an outward-facing endometrial epithelial surface in organoid culture. The demonstrated ability to modify the polarity of EEO without impairing their functionality, coupled with successful co-culture with microbial and embryonic entities, paves the way for investigating complex interactions at the endometrial epithelial surface at scale. These insights hold promise for elucidating physiological processes such as embryo implantation and pathogenic processes like infection. Future research can leverage this model to gain a deeper understanding of these interactions, potentially unveiling new therapeutic strategies for conditions related to the endometrial epithelium.

## Supplementary Materials

Supplementary Figure 1. (A) Immunofluorescence staining of F-Actin (grey) on paraffin sections of AI or AO human endometrial epithelial organoids. Organoids were counterstained with Hoechst (blue); scale bars, 20 µm. (B) Representative IF images of AI or AO showing, β-catenin (red) on paraffin sections. Scale bars, 40 µm. (C) Representative IF images of KI67 (red) and ZO1 (green) on paraffin sections of AI or AO human endometrial epithelial organoids. Organoids were counterstained with Hoechst (blue); scale bars, 40 µm (i) and 80 µm (ii). (D) Immunostaining staining of FOXA2 (red) and ZO1 (green) on paraffin sections of AI or AO. Organoids were counterstained with Hoechst (blue); scale bars, 40 µm. (E) Organoids generated from additional independent donors at day 8 of suspension culture; immunofluorescence staining of ZO1 (green) demonstrated polarity reversal. Organoids were counterstained with Hoechst (blue). Scale bars, 40 µm. (F) Polarity reversal of organoids is detected with SEM in suspension culture for 0, 1, 4 and 8 days; scale bars, 100 µm. Areas outlined in boxes are magnified to demonstrate the distribution of microvilli on apical surface; scale bars, 20 µm. (G) Bright-field images of mouse organoids grown in cultrex BME or in suspension culture for 4 days; scale bars, 100 µm (i & iii). Representative images of immunofluorescence staining of ZO1 on AI or AO mouse organoids and counterstained with Hoechst (ii & iv); scale bars, 20 µm (ii) and 40 µm (iv).

Supplementary Figure 2. Apical-out organoids exhibit robust response to estrogen. (A) Representative images of immunofluorescence staining of ESR1 (red) and ZO1 (green) on paraffin sections of AI and AO treated with vehicle or E2. Organoids were counterstained with Hoechst (blue) (i-ii & iv-v); scale bars are 20 µm (i-ii) and 40 µm (iii-iv). Quantification of ESR1-positive cells (iii-iv). (B) Representative images of immunofluorescence staining of KI67 (red) and ZO1 (green). Organoids were counterstained with Hoechst (blue) (i-ii & iv-v); scale bars are 20 µm (i-ii) and 40 µm (iii-iv). Quantification of KI67-positive cells (iii-iv). (C) Quantification of length of microvilli and number of cili in AI and AO treated with vehicle or E2 or E2+MPA. Plots in A-C represent mean ± SD. Datapoints on the plots represent the different organoids. p <0.01= **, p <0.001= ***, p <0.0001= **** (Student’s t test).

## Declaration of interest

The authors declare that there is no conflict of interest that could be perceived as prejudicing the impartiality of the research reported.

## Funding

This work was supported by NIH Grant R01HD096266 and U01HD104482 from the Eunice Kennedy Shriver National Institute of Child Health and Development (TES), and new faculty startup funds from the University of Missourihttp://dx.doi.org/10.13039/100007165-Columbia (AMK and BPhttp://dx.doi.org/10.13039/100004364T).

## Author contribution statement

VA, TES, and AMK designed research; VA, SGY performed research; BT and TES provided critical reagents; VA and AMK analyzed data; and VA and AMK wrote the manuscript. All authors edited and approved the final manuscript.

## Competing interest statement

BT is a founding member and chief scientific officer at RenOVAte Biosciences Inc. All other authors declare no competing interest.
