# Supplementary figures and images for: Development of polarity-reversed endometrial epithelial organoids
Source: Reproduction. 2024 Feb 15;167(3):e230478. doi: 10.1530/REP-23-0478 (PMC10959009; doi:10.1530/REP-23-0478)

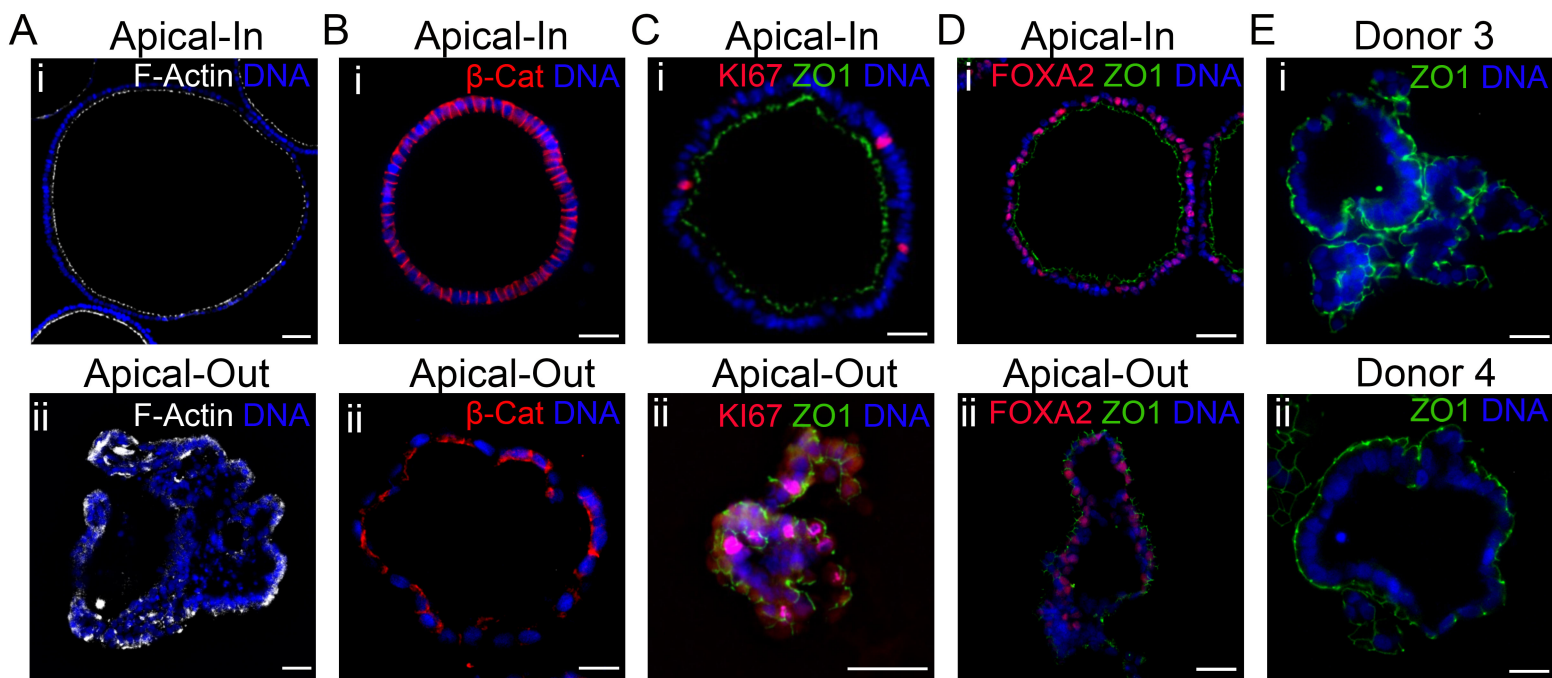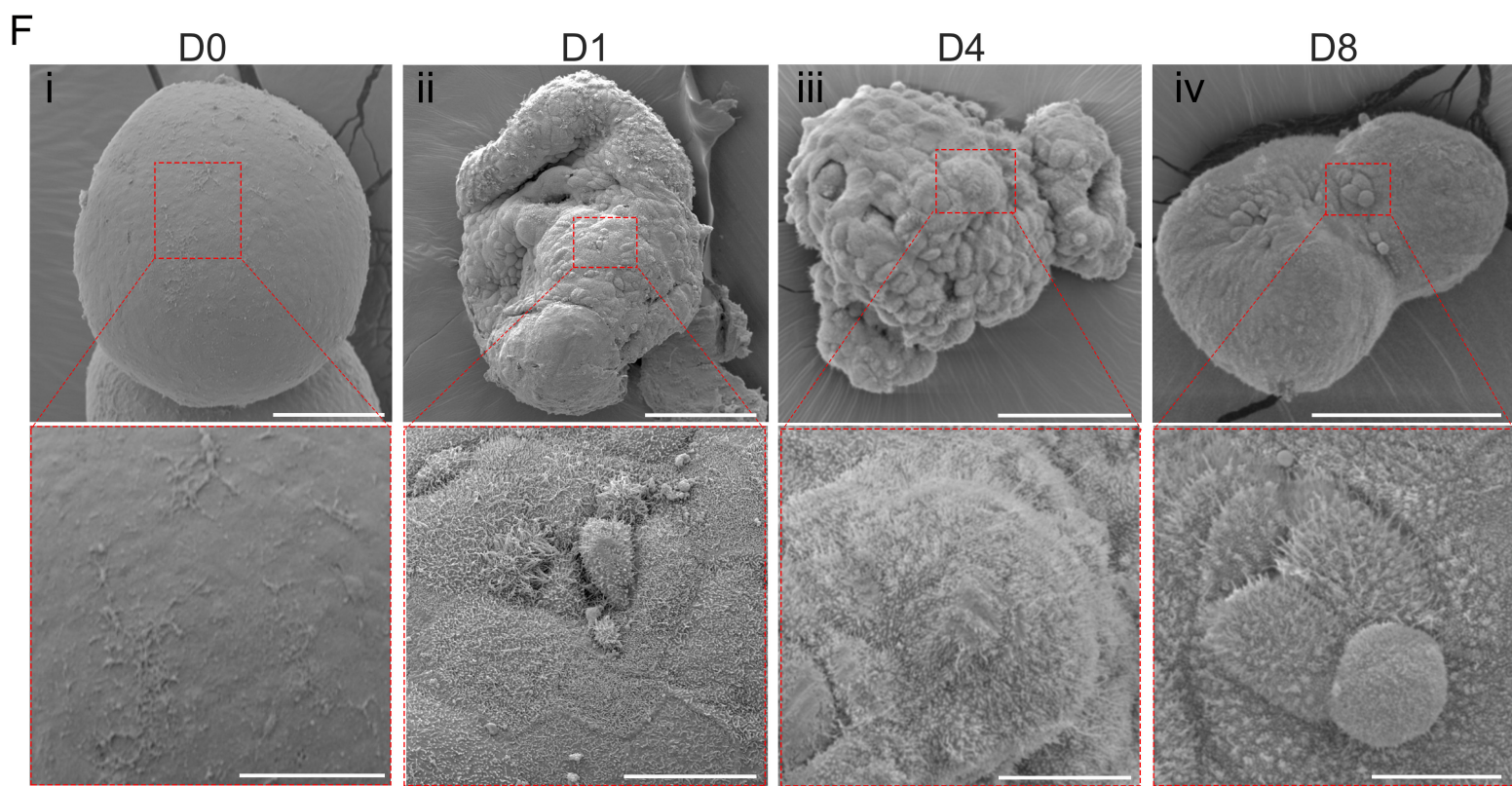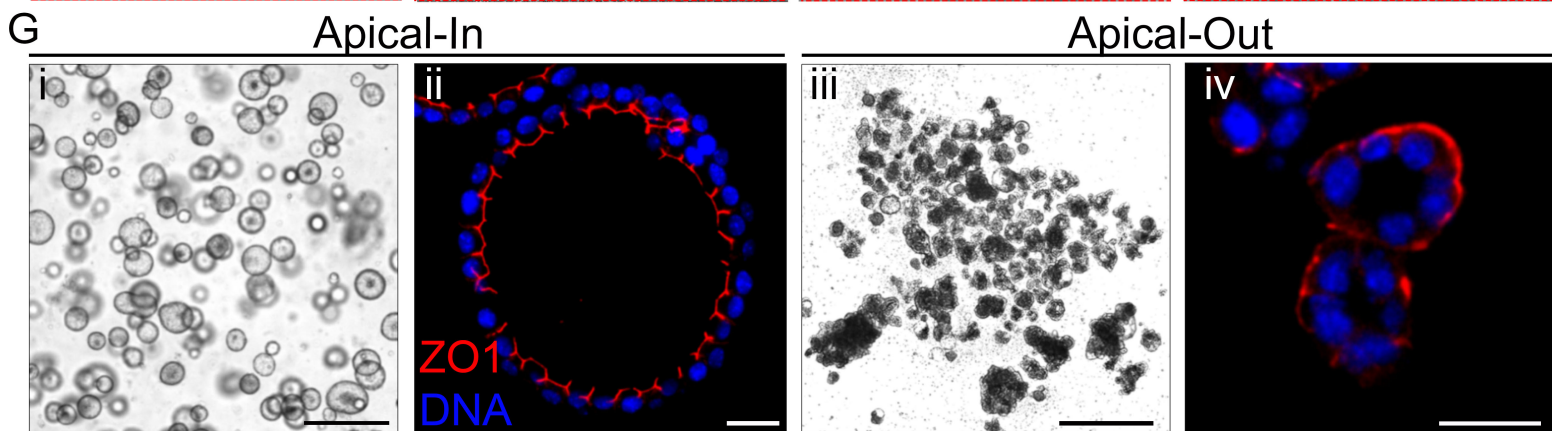

Supplement: Supplementary Figure 1. (A) Immunofluorescence staining of F-Actin (grey) on paraffin sections of AI or AO human endometrial epithelial organoids. Organoids were counterstained with Hoechst (blue); scale bars, 20 µm. (B) Representative IF images of AI or AO showing, β-catenin (red) on paraffin secti [file supplementary_figure_1.pdf]

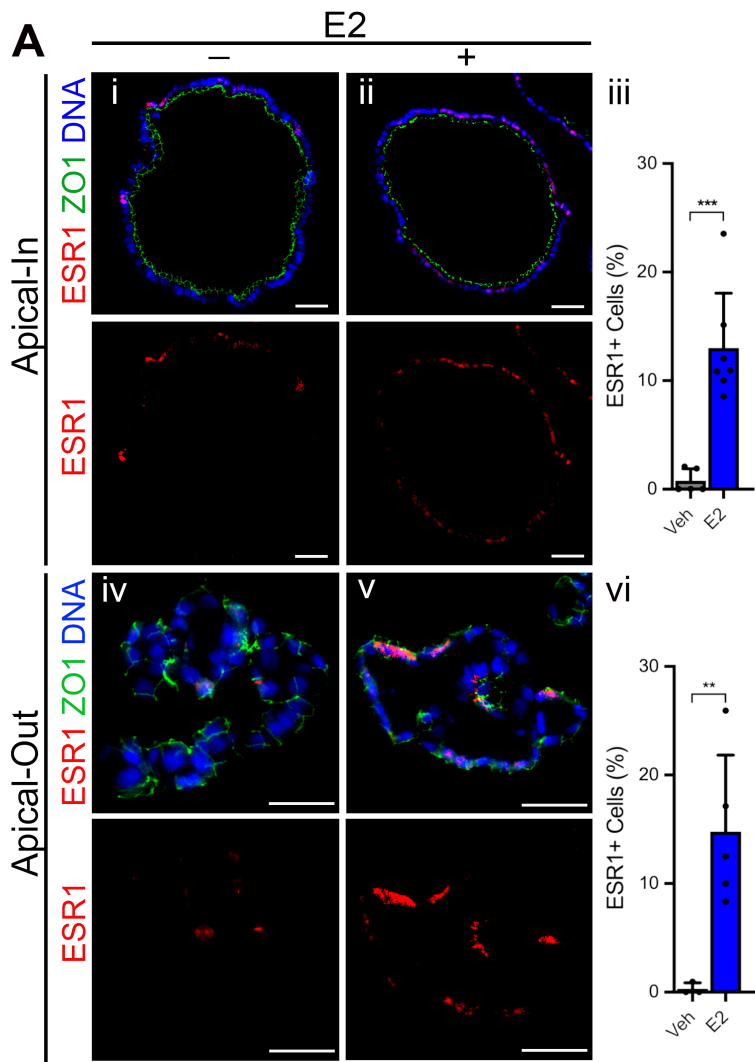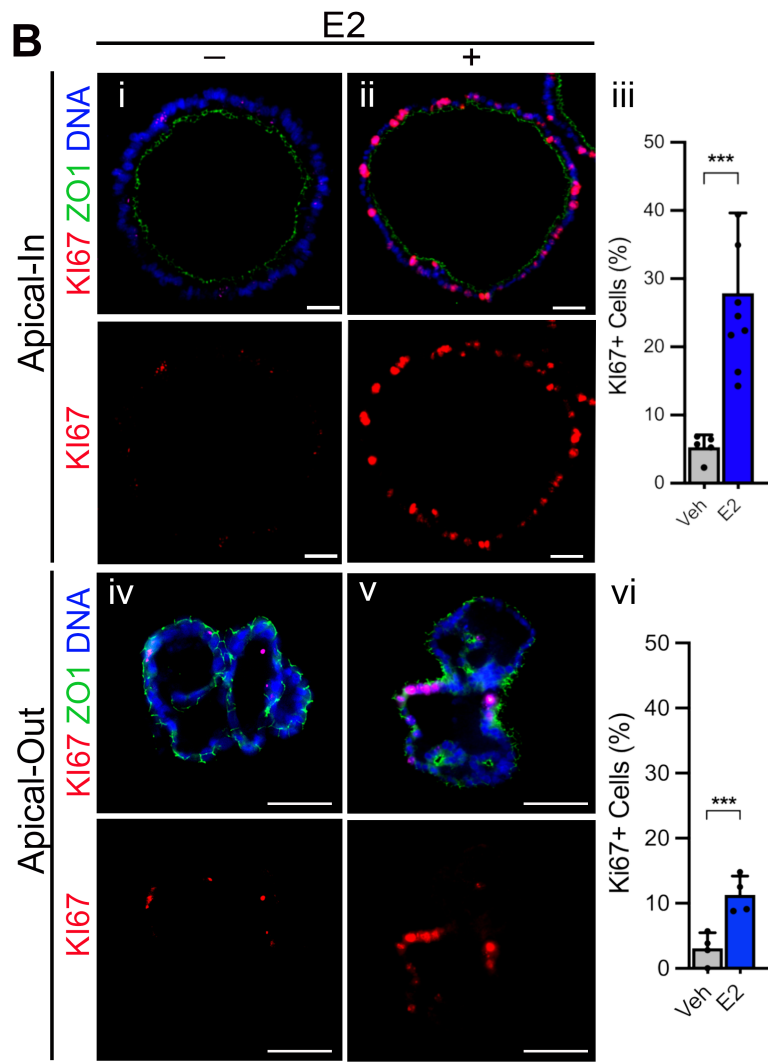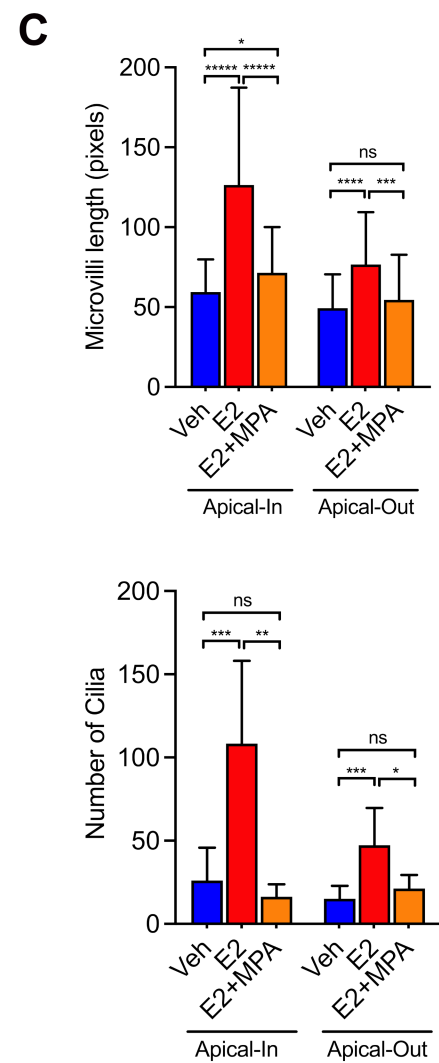

Supplement: Supplementary Figure 2. Apical-out organoids exhibit robust response to estrogen. (A) Representative images of immunofluorescence staining of ESR1 (red) and ZO1 (green) on paraffin sections of AI and AO treated with vehicle or E2. Organoids were counterstained with Hoechst (blue) (i-ii & iv-v); scal [file supplementary_figure_2.pdf]
